# Impact of potassium test sample rejections on routine laboratory service, South Africa

**DOI:** 10.4102/ajlm.v12i1.2239

**Published:** 2023-12-22

**Authors:** Sarah McAlpine, Bettina Chale-Matsau

**Affiliations:** 1 Department of Chemical Pathology, National Health Laboratory Service, Pretoria, South Africa; 2 Department of Chemical Pathology, Faculty of Health Sciences, University of Pretoria, Pretoria, South Africa

**Keywords:** hyperkalaemia, pseudohyperkalaemia, pre-analytical factors, result rejection, sample rejection

## Abstract

**Background:**

Accurate potassium measurements are necessary for effective clinical management of hyperkalaemia. Pre-analytical factors may affect laboratory measurements, leading to erroneous results and inappropriate patient management and negatively impact the efficiency and finances of laboratories and hospitals.

**Objective:**

This study evaluated the impact of rejected potassium test requests on laboratory service.

**Methods:**

We conducted a retrospective descriptive study to assess potassium test data at a public laboratory in Pretoria, Gauteng, South Africa, using samples collected from an academic hospital, peripheral hospitals, and outpatient clinics between January 2018 to December 2018. We assessed the relationship between reasons for rejection and health facility type, as well as financial implications for the laboratory.

**Results:**

The potassium result rejection rate was 15.1% (29 806 samples), out of the 197 405 requests received. The most common reasons for rejection were old sample (> 1 day old) (41.4%; 12 348 rejections) and haemolysis (38.2%; 11 398 rejections). The most frequent reason for rejections at the central, academic hospital was haemolysis (42.0%), while old sample was the most common reason for rejection at peripheral hospitals (43.4%; 4119/9493 requests) and outpatient health facilities (57.2%; 7208/12 605 requests) (*p* = 0.022). The total cost of potassium sample rejection over the study period was substantial, given the resource constraints in this setting.

**Conclusion:**

Peripheral hospitals and outpatient departments accounted for the majority of rejected potassium testing results, possibly resulting from delays in transportation; causing substantial financial impact on the laboratory. Improved sample collection, handling, and expedited transportation are recommended.

**What this study adds:**

This study highlights the importance of appropriate sample collection and handling and the undesirable consequences of non-adherence to these pre-analytical considerations.

## Introduction

Hyperkalaemia is defined as potassium levels greater than 5.5 mmol/L.^1^ It can occur following dysfunction of the renin-angiotensin-aldosterone system due to kidney failure, adrenal hypofunction, or the use of certain drugs, thus resulting in decreased potassium elimination.^2,3^ Other common causes of hyperkalaemia include increased release of potassium from cells, as seen in diabetic ketoacidosis due to decreased insulin levels, as well as cellular breakdown or necrosis, such as rhabdomyolysis, tumour lysis syndrome, haemolysis, trauma, and burns.^4,5^ Hyperkalaemia is also not unusual in patients with thrombocytosis or leucocytosis.^5,6^ Persistent hyperkalaemia can be life-threatening, causing cardiac arrhythmias with possible cardiac arrest or paralysis of the respiratory muscles even with slight deviations from normal levels (3.5 mmol/L – 5.0 mmol/L).^1,6^ Thus, accurate laboratory potassium measurements are necessary for clinicians to make appropriate medical decisions.

Within the laboratory, the quality and confidence of a test result depends on the quality and functioning of the total testing process, which encompasses the pre-analytical, analytical and post-analytical stages.^7,8^ This is important as 60% – 70% of medical decisions rely on laboratory investigations.^9^ Due to advancements in laboratory methods, instruments, and monitoring through internal quality control and external quality assurance programmes, fewer errors are typically attributed to the analytical phase.^8,10^ The pre-analytical phase involves test requisition and sample labelling, collection, handling, transport, and processing. This phase accounts for up to 70% – 75% of all laboratory errors reported, most of which are due to human errors.^8,9^ These errors can lead to samples that are unsuitable for analysis or unreportable results requiring rejection. Pre-analytical factors that may affect sample quality include haemolysed, clotted, or icteric samples, as well as samples that are mislabelled, unlabelled, or collected in inappropriate tubes. Insufficient sample volume not only limits the number of tests that can be done from the received sample but can also affect sample quality, as the blood-to-additive ratio may interfere with analysis and produce erroneous results.^8,9^ Mild contamination by ethylenediaminetetraacetic acid (EDTA-K), a potassium-containing anticoagulant, may also cause subtle analyte changes that can be missed.^11^ Prolonged centrifugation of samples during the pre-analytical phase can also interfere with sample quality where, for example, platelets can lyse, resulting in falsely elevated potassium levels.^12,13,14^

Falsely elevated potassium levels or pseudohyperkalaemia occurs when generated results are not consistent with the clinical features of the patient.^4,12^ Not only can pseudohyperkalaemia result in inappropriate patient management, but it can also mask hypokalaemia, leading to a missed diagnosis as concentrations may falsely present within a ‘normal’ reference interval.^12^ The most commonly occurring source of pseudohyperkalaemia is haemolysis, which is reported to cause up to fivefold more rejections than any other reason.^7,13^

A subset of haemolysed samples may result from endogenous causes such as haemolytic anaemia, immune reactions, toxin exposure, and haemodialysis treatment, or due to direct damage to red blood cells.^3,5^ In vitro or exogenous haemolysis accounts for most haemolysed cases. Causes include poor sample acquisition technique, substandard handling and transport, prolonged storage time, and delayed processing.^15,16^ Sample acquisition techniques that specifically affect potassium include prolonged tourniquet application and patient fist clenching, which can cause a 1 mmol/L – 2 mmol/L increase in potassium levels due to potassium efflux from cells during muscle contraction.^12,13,14^ The use of needles with unsuitable diameters during sample collection can also result in haemolysis. In addition, cold temperatures can decrease the function of the sodium/potassium-adenosine triphosphase pump, resulting in the passive movement of potassium down the concentration gradient and out of red blood cells.^12^

Rejection of samples or test results inconveniences both patients and healthcare personnel as it delays urgent clinical decision-making. Specimen recollection may be required, leading to prolonged admission for the patient and unnecessary costs to the laboratory and the hospital or clinic.^10,17^ Pre-analytical errors are estimated to cost approximately 0.23% – 1.02% of a hospital’s total operating budget in the United States.^9,10^ While there are at present no local studies on the financial implications of hyperkalaemia on the health sector in South Africa, the prevalence of hyperkalaemia^18^ and its contribution to prolonged hospital stay have been previously described.^19^ Magwai and colleagues recently described a rejection rate of 1.4% in an academic laboratory in South Africa.^20^

Although sample and result rejection may appear to be a small and insignificant factor in the total testing process, it is an important quality indicator for laboratories and can have great implications for the patient and health facility. This study thus aimed to assess the frequency and reasons for potassium test request rejection at the National Health Laboratory Service (NHLS), Tshwane Academic Division (TAD), South Africa, during the period from 01 January 2018 to 31 December 2018 and the financial impact of these rejections on this laboratory.

## Methods

### Ethical considerations

This study was approved by the University of Pretoria Research Ethics Committee (number 578/2020). Patient consent was waived by the ethics committee as the study was based on retrospective data. Patient details such as name, address, hospital number or unique laboratory identification numbers were not used to ensure patient confidentiality.

### Study design and setting

A retrospective descriptive observational study was conducted using data for potassium test requests, including both resulted and rejected tests, at the NHLS TAD Chemistry laboratory from 01 January 2018 to 31 December 2018. These data were requested from the NHLS data warehouse and were on samples from facilities in Pretoria, Gauteng, South Africa. Variables considered were reasons for rejection and the facility where the sample was obtained. Rejected potassium test requests were either for unreported results or samples not tested for potassium, each of which was replaced by a reason for rejection. Potassium requests generally form part of a urea and electrolytes profile. The NHLS TAD is a tertiary laboratory located in the 832-bed, Steve Biko Academic Hospital. The laboratory also receives samples from six peripheral hospitals (PH) and 264 outpatient clinics located at average distances of 60 km and 30 km from the laboratory. The NHLS has small laboratories based at PHs. These small laboratories are mostly closed after work hours, during which test requests are referred to TAD. The laboratory receives approximately 350 000 all-inclusive test requests per month, tested using the Abbott Architect C8000 chemistry analyser (Abbott Laboratories, Chicago, Illinois, United States). We excluded all data for which patient location was not recorded as well as samples registered at peripheral laboratories and referred to TAD.

### Data analysis

We evaluated data on rejected potassium test results from three categories of health facilities to determine the frequency of result rejection and reasons for rejection, and to estimate the financial loss incurred by the laboratory as a result of these rejections. Financial loss was estimated based on the NHLS cost-per-test charge (potassium cost at R30.35 South African rand [ZAR] per test) for 2018 (study period). Data analysis and descriptive statistics were computed using Microsoft Excel 2016 (Microsoft Corporation, Redmond, Washington, United States) spreadsheets. The R statistical software version 3.6.0 (R Foundation, Indianapolis, Indiana, United States) was used for statistical calculations.^21^ Categorical data were expressed as frequencies and percentages. We compared the distribution of rejection reasons between Steve Biko Academic Hospital (a central academic hospital), PHs and outpatient health facilities using the Chi-square test. *P*-values less than 0.05 were considered statistically significant.

## Results

### Potassium test result rejection rate and reasons for rejection

**FIGURE 1:**
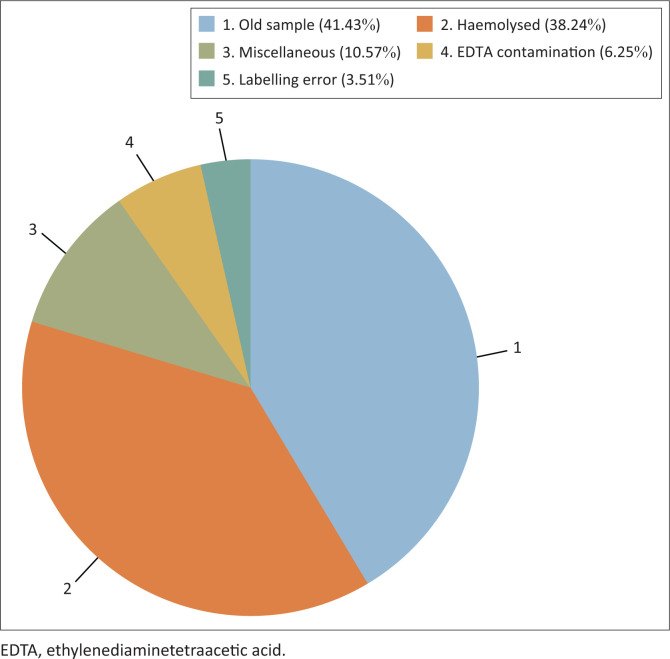
Reasons for rejection of potassium test requests at the National Health Laboratory Service, Tshwane Academic Division, Gauteng, South Africa, January 2018 – December 2018.

The total number of potassium tests requested during the study period was 197 405 (Table 1), of which 167 599 were resulted and 29 806 were rejected (15.1%). The most common reasons for rejection were old samples (1 day or older) (41.4%; *n* = 12 348 rejections), haemolysed samples (38.2%; *n* = 11 398 rejections), EDTA-K contamination (which was based on decreased magnesium, calcium, and alkaline phosphatase results) (6.3%; *n* = 1864 rejections), and labelling errors (3.5%; *n* = 1046 rejections), which included missing or mismatched information on the sample and the request form (Figure 1). Other rejection reasons, accounting for 10.6% (*n* = 3150) of all rejections, were grouped as miscellaneous reasons and included ‘leaked’, ‘wrong tube’, ‘lost in transit’, ‘insufficient specimen’, ‘specimen container broken’, ‘duplicate registration’, and ‘specimen not received’.

**TABLE 1:** Number of potassium test requests and rejections by source facility category. National Health Laboratory Service, Tshwane Academic Division, Gauteng, South Africa, January 2018 – December 2018.

Facility category	Number of facilities	Number of requests	Number of rejections	Facility rejection percentage (%)	Total sample rejection percentage (%)
Outpatient clinics	264	35 699	12 605	35.3	42.3
Peripheral hospitals	6	41 019	9493	23.1	31.9
Academic hospital	1	120 687	7708	6.4	25.9

### Result rejections based on facility type

Rejected potassium test requests were from a total of 271 facilities, which were divided into three categories. These were Steve Biko Academic Hospital, which is the central academic hospital, PHs (*n* = 6), and outpatient health facilities (*n* = 264). Outpatient health facilities made up 42.3% of the total number of rejected results, PHs made up 31.9%, and Steve Biko Academic Hospital alone made up 25.9% of all potassium result rejections in the specified study period.

**FIGURE 2:**
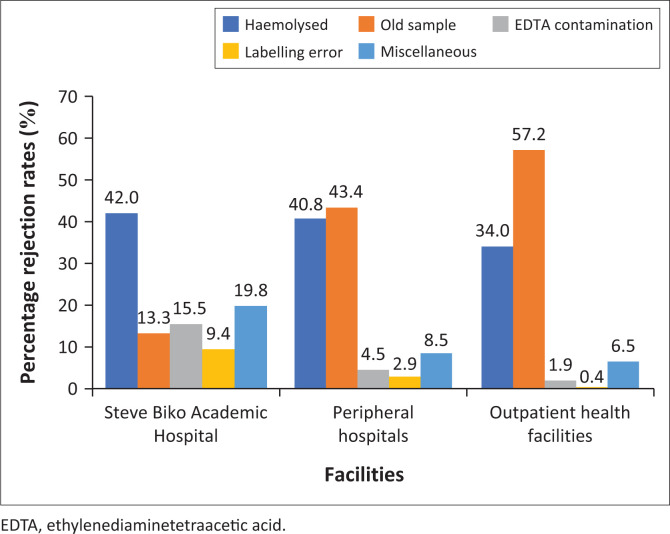
Reasons for rejection of potassium test requests at the National Health Laboratory Service, Tshwane Academic Division, Gauteng, South Africa, January 2018 – December 2018.

Haemolysed sample, which caused 42.0% of rejections, was the most reported reason for rejection from Steve Biko Academic Hospital (Figure 2). This was followed by miscellaneous reasons for rejection (19.8%) and EDTA-K contamination (15.5%). This differed from the PHs and the outpatient health facilities where old sample was the most common cause of rejection (43.4% and 57.2%), followed by haemolysed samples (40.78% and 34.0%). There was a statistically significant difference (*p* = 0.022) between the reasons for potassium result rejection at the different facility types (Table 2).

**TABLE 2:** Observed and expected values for number of potassium test rejections by source facility category. National Health Laboratory Service, Tshwane Academic Division, Gauteng, South Africa, January 2018 – December 2018.

Reasons for rejection	Facility type						Total
	Steve Biko Academic Hospital		Peripheral hospitals		Outpatient clinics and hospitals		
	Observed value	Expected value	Observed value	Expected value	Observed value	Expected value	
Haemolysed	3239	2947.59	3869	3630.18	4290	4820.23	11 398
Old sample	1021	3193.26	4119	3932.75	7208	5221.99	12 348
EDTA contamination	1192	482.04	429	593.67	243	788.29	1864
Labelling error	728	270.50	272	333.14	46	442.35	1046
Miscellaneous	1528	814.61	804	1003.25	818	1332.14	3150

Note: Observed and expected values were used in the calculation of the Pearson’s Chi-squared test. Overall *p*-value = 0.022.

EDTA, ethylenediaminetetraacetic acid.

### Financial impact of potassium test results rejection

**FIGURE 3:**
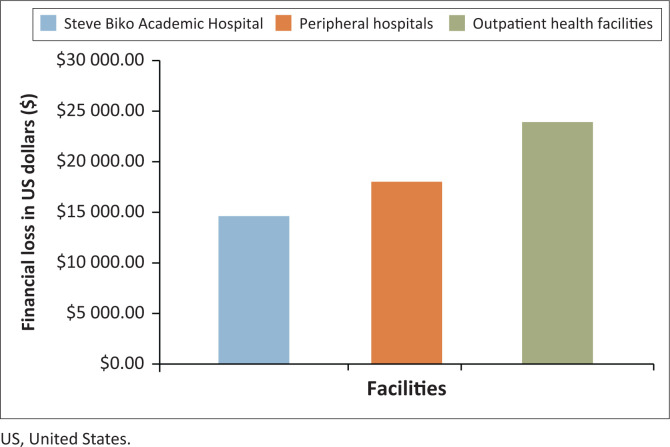
The estimated financial loss incurred by the National Health Laboratory Service, Tshwane Academic Division, Gauteng, South Africa due to potassium test request rejections from Steve Biko Academic Hospital, peripheral hospitals, and outpatient health fa cilities, January 2018 – December 2018.

The NHLS cost per test for potassium testing as per the 2018 price list was approximately $1.90 United States dollars (USD; equivalent to R30.35 ZAR). With a total of 29 806 rejected potassium test requests, the total cost to the laboratory was approximately $56 000.00 USD (R986 876.00 ZAR) in 2018 (Figure 3).

## Discussion

The potassium result rejection rate for the NHLS TAD Chemistry laboratory in 2018 was 15.1%, and there was a statistically significant difference between the reasons for potassium result rejection at the different facility types. Old sample was the main reason for rejections, comprising 41.4% of total rejections. This was the most common reason for rejection for both PHs and outpatient facilities. Owing to the location of some of these facilities, it is likely that the reasons for delayed analysis might be multifactorial. These are likely to include distance, long time lapse following collection, storage, and transportation conditions. Other potential reasons for delay in testing may include batch testing or factors related to the testing laboratory itself such as excessive workload, instrument issues, and sample sharing between departments, which may delay sample arrival to the chemistry laboratory as previously described in studies from China and the United States.^16,22^

Old samples are rejected because of biochemical changes that occur as red blood cells break down during long storage, thus leading to erroneous results.^15^ Delays in sample analysis can affect accuracy and quality, and, as indicated in a study from the United States, samples stored at temperatures of 2 °C – 8 °C for longer than 24 h result in spuriously elevated potassium levels due to cessation of the sodium/potassium-adenosine triphosphatase pump.^12^ A study from France has indicated that although potassium levels are stable at room temperature, samples should be analysed within 4 h – 6 h for accurate measurements.^23^ Moreover, increased potassium levels have been reported in samples stored at room temperature within 1 h of collection in a study done in China in 2017.^16^ Another factor to consider when samples are sent to external or distant laboratories for testing is whether the samples were centrifuged at the collection site prior to transportation or only after they arrived at the testing laboratory. Delayed serum separation may cause potassium to leak out of the red blood cells, yielding hyperkalaemia. Ideally, according to a United Kingdom study done in 2003, samples should be centrifuged within 1 h of collection to prevent these falsely increased potassium levels.^24^ Additionally, an important aspect to be aware of for potassium measurements is susceptibility to seasonal pseudohyperkalaemia, where potassium levels are elevated in the cooler winter months owing to the inhibition of the sodium/potassium-adenosine triphosphatase pump activity.^12,24^

Interestingly, the main reason for result rejection in this study, old sample, is not the most common cause of rejection reported in previous studies done in Ethiopia, the United States, and Turkey between 2015 and 2016.^10,11,25,26^ The major reasons for rejection reported in these studies include haemolysis, insufficient sample, incorrect sample or tube, clotted samples, contamination, and mislabelled or unlabelled samples.^26,27^ This difference may, however, be explained by the fact that about 74% of all samples received at this laboratory were referred from off-site facilities, thus making delayed sample analysis a major factor causing rejection.

The second most common reason for potassium result rejection in this study was haemolysed samples (38.2%). This was the main reason for potassium result rejection at the Steve Biko Academic Hospital (42.02%), a facility that makes up a quarter of all samples received by the laboratory. At the peripheral facilities, approximately 50% of rejections were due to haemolysed samples, which is lower than previous reports from Australia and Turkey in 2010, where haemolysed samples account for about 60% of all rejections.^6,28^ Findings from previous studies done in Italy in 2008 and Malaysia in 2019 suggest that haemolysis is the main cause of pseudohyperkalaemia.^7,13^ Making medical decisions based on these erroneous results can lead to inappropriate patient care. This may occur when there is pseudohyperkalaemia or in instances when hypokalaemia is missed due to false potassium elevation in the sample.^12^ A Malaysian study done in 2019 demonstrated that although haemolysis may occur in vivo, this only occurs in about 2% of cases.^7^ When in vivo haemolysis is suspected, additional information such as patient history, haptoglobin measurements, bilirubin levels, and red blood cell count are required to arrive at the diagnosis.^7^ In 2020, a study in Italy reported that in vitro or exogenous causes are more common and include poor sample collection techniques, poor sample handling and transport, prolonged storage time and inadequate storage temperature.^29^ Personnel collecting samples should be trained and educated on the importance of correct collection techniques. A study investigating the impact of educational training among nurses in a hospital in Oman in 2017 showed about 75.9% improvement in sample quality after re-training.^30^ The availability of specially trained phlebotomy personnel within a facility is important not just for proper blood sample collection, but also to ease the workload on the already overworked nurses and doctors, who may tend to rush sample collection due to the increased workload.^30^ Haemolysed samples certainly pose a serious challenge for clinical laboratories.^7^

Contamination by EDTA-K anticoagulant as a cause of pseudohyperkalaemia accounted for 6.3% of the potassium rejections in this study and is common in clinical chemistry laboratories.^31^ As reported in studies from Thailand in 2019^32^ and India in 2020,^33^ EDTA-K contamination may potentially lead to patient mismanagement. Thus, technique and order of draw need to be cautiously considered during sample collection to avoid contamination.^34^

Rejection of test results can negatively impact patient care at the health facility and laboratory. Importantly, rejected results can delay critical and potentially life-saving clinical decisions. Based on our calculations, the NHLS TAD incurred a financial loss of almost R1 000 000.00 ZAR (approximately $56 000.00 USD) in a single year due to potassium test request rejections. An additional aspect to consider is the person-hours wasted due to these rejections. Rejection of 29 806 potassium test requests over a 12-month period equates to approximately 2500 rejections per month. If the processing time of each of these samples is 10 min, 25 000 min or 416 h per month are lost on wasted labour. This equates to approximately 17 days per month. Also, additional time is wasted as laboratory staff remove erroneous results and assign reasons for rejection. To solve this, the NHLS TAD could implement automatic cancellation by the laboratory information system of potassium requests that are older than 1 day. This would prevent the sample from being analysed and eliminate the subsequent wastage of financial resources and labour.

### Limitations

Access to data was mainly limited to NHLS archived data with no knowledge how the samples were collected or stored. The rejection data comprised mostly potassium requests that were either of unreported results or samples not tested for potassium, each of which was replaced by a reason for rejection and could therefore not be reported. The cost of these rejections to the hospitals and the effect on patient management could not be quantified as no clinical information was available for this estimation. The cost could only be estimated for the laboratory and could not be done for the health facilities.

### Conclusion

Findings from this study have shown a high rate of rejection of potassium test requests across all facilities, with PHs and outpatients accounting for most of the rejected potassium test requests. Old sample was the most common reason in facilities located off site due to delays in sample processing. As indicated in the study, test rejections can have significant financial implications for the laboratory.
